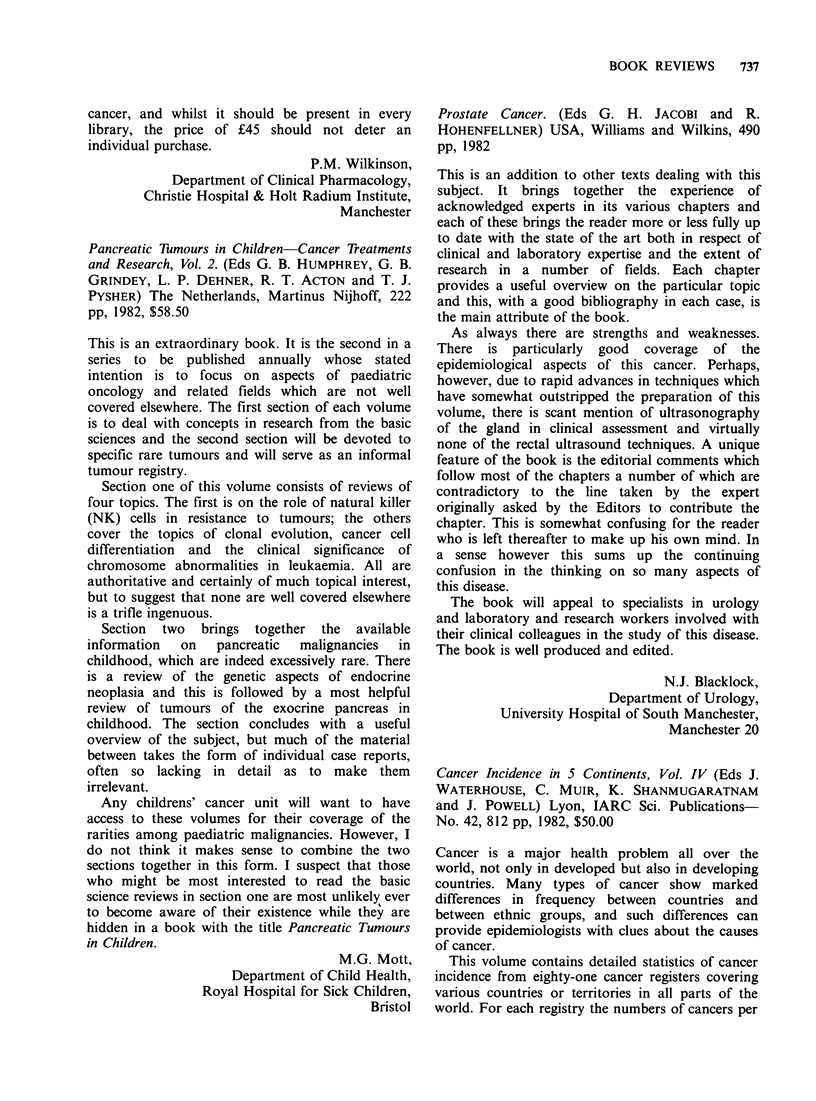# Pancreatic Tumours in Children—Cancer Treatments and Research

**Published:** 1983-05

**Authors:** M.G. Mott


*Pancreatic Tumours in Children—Cancer Treatments and Research, Vol. 2.* (Eds G. B. HUMPHREY, G. B. GRINDEY, L. P. DEHNER, R. T. ACTON and T. J. PYSHER) The Netherlands, Martinus Nijhoff, 222 pp, 1982, $58.50

This is an extraordinary book. It is the second in a series to be published annually whose stated intention is to focus on aspects of paediatric oncology and related fields which are not well covered elsewhere. The first section of each volume is to deal with concepts in research from the basic sciences and the second section will be devoted to specific rare tumours and will serve as an informal tumour registry.

Section one of this volume consists of reviews of four topics. The first is on the role of natural killer (NK) cells in resistance to tumours; the others cover the topics of clonal evolution, cancer cell differentiation and the clinical significance of chromosome abnormalities in leukaemia. All are authoritative and certainly of much topical interest, but to suggest that none are well covered elsewhere is a trifle ingenuous.

Section two brings together the available information on pancreatic malignancies in childhood, which are indeed excessively rare. There is a review of the genetic aspects of endocrine neoplasia and this is followed by a most helpful review of tumours of the exocrine pancreas in childhood. The section concludes with a useful overview of the subject, but much of the material between takes the form of individual case reports, often so lacking in detail as to make them irrelevant.

Any childrens' cancer unit will want to have access to these volumes for their coverage of the rarities among paediatric malignancies. However, I do not think it makes sense to combine the two sections together in this form. I suspect that those who might be most interested to read the basic science reviews in section one are most unlikely ever to become aware of their existence while they are hidden in a book with the title *Pancreatic Tumours in Children.*

M.G. Mott,
Department of Child Health,
Royal Hospital for Sick Children,
Bristol